# Improved clinical outcomes and a low rate of failure following implantation of a patellofemoral inlay arthroplasty model featuring an enlarged lateral offset – a prospective clinical and radiographic evaluation at short term follow-up

**DOI:** 10.1007/s00402-025-05832-w

**Published:** 2025-03-22

**Authors:** Matthias Cotic, Tiago Martinho, Svenja Höger, Marco-Christopher Rupp, Maximilian Hinz, Sebastian Siebenlist, Andreas B. Imhoff, Armin Runer

**Affiliations:** 1 Department of Sports Orthopaedics, Technical University of Munich, Ismaninger Str. 22, 81675 Munich, Germany; 2 Faculty of Medicine, University of Geneva, Geneva, Switzerland

**Keywords:** Knee, Patellofemoral osteoarthritis, Patellofemoral arthroplasty, Patellofemoral inlay arthroplasty, HemiCap kahuna

## Abstract

**Purpose:**

To prospectively evaluate clinical, functional, radiographic, and sports-related short-term outcomes following isolated patellofemoral inlay arthroplasty (PFIA) utilizing an inlay arthroplasty model featuring an enlarged lateral offset.

**Methods:**

Patients who underwent patellofemoral inlay arthroplasty (PFIA) with the Hemi-CAP^®^ Kahuna Prosthesis (Anika Therapeutics, Franklin, MA, USA) between January 2017 and July 2020 were included in the study and assessed both preoperatively and at a minimum follow-up of 24 months postoperatively. Patient-reported outcomes measures (PROs) included the transformed Western Ontario and McMaster Universities Arthritis Index (WOMAC), Visual Analogue Scale (VAS) for pain, and Tegner Activity Scale. The Kellgren-Lawrence grading scale was used to assess tibiofemoral osteoarthritis (OA) progression. The Caton-Deschamps Index was used to assess differences in pre- to postoperative patellar height.

**Results:**

Eighteen patients (19 knees, 86% follow-up) were available at 28.2 ± 9.1 (range 24.0–55.0) months. WOMAC score (55.8 ± 16.0 to 77.2 ± 17.0; *p* <.001) and VAS for pain (6.1 ± 2.3 to 2.7 ± 2.1; *p* <.001) improved statistically significantly from pre- to postoperative whereas the mean Tegner Activity Scale (2.5 ± 1.3 to 3.1 ± 1.3; *p* >.05) improved slightly. No significant progression of tibiofemoral OA (*p* >.05) or changes in patellar height (*p* >.05) were observed. No implant-related maltracking or patellar instability was reported. One patient (5.3%) required revision surgery due to aseptic component loosening.

**Conclusion:**

Isolated patellofemoral inlay arthroplasty (PFIA) utilizing an implant with an enlarged lateral offset has been shown to be an effective and safe intervention for patients with symptomatic patellofemoral osteoarthritis. The procedure significantly improved knee function and pain relief, with low failure rates observed at short-term follow-up.

**Level of evidence:**

4, prospective case series.

## Introduction

In patients with isolated patellofemoral osteoarthritis (OA), patellofemoral inlay arthroplasty (PFIA) is considered a viable treatment option with good clinical results and survival rates [12, 18, 24]. In particular, the PFIA models, which offer the opportunity to perform individualized anatomic trochlear resurfacing, have garnered recent attention [5, 13, 14].

However, several implant-related drawbacks have been recognized. A multicenter study reported that the geometry of the implant contributed to postoperative failures in patients with patellofemoral instability [14]. Additionally, an independent study found that approximately 50% of the patients required revision surgery within two years after surgery due to exacerbated pain and the occurrence of a “clunk” phenomenon [1]. Concerns were raised regarding the implant’s inability to adequately guide the patella during full extension, as it only provides coverage for the central region of the trochlea while leaving the cranio-lateral area unsupported [1, 10].

To address these limitations, a new inlay trochlear component (Hemi-CAP^®^Kahuna, Anika Therapeutics, Franklin, MA, USA) was developed featuring an enlarged lateral offset aimed at enhancing patellofemoral tracking. While the theoretical advantages of this design appear intuitive, no clinical results have been reported yet.

The purpose of this study was to prospectively evaluate clinical, functional, radiographic, and sports-related outcomes following isolated PFIA utilizing a trochlear implant featuring an enlarged lateral offset, with a minimum follow-up period of 24 months.

It has been hypothesized that isolated PFIA using the new implant provides good postoperative clinical and functional outcomes with low failure- and complication rates.

## Materials and methods

This study was approved by the institutional review board of the Technical University of Munich (registration number 2022-200-S). All patients gave their written informed consent prior to study inclusion. Patients undergoing primary, isolated PFIA using the inlay trochlear component featuring an enlarged lateral offset performed at a single specialized orthopaedic center between 01/2017 and 07/2020 were recorded in a prospectively administered database and included in the study. Exclusion criteria included any additional procedures performed to address patellofemoral instability, patellofemoral or tibiofemoral malalignment, or revision surgery.

Surgery was indicated in patients with isolated disabling high-grade patellofemoral OA (grade III or higher according to Kellgren-Lawrence [15]) or high-grade patellofemoral chondral defects (grade III-IV according to Outerbridge [19]) refractory to conservative treatment and/or prior surgery. Contraindications for PFIA were symptomatic tibiofemoral OA with pain during activities of daily living, systematic inflammatory arthropathy, chondrocalcinosis, chronic regional pain syndrome, active infection, and fixed loss of knee range of motion. Isolated PFIA was performed in patients without patellofemoral instability, patellofemoral malalignment defined as tibial tuberosity trochlear groove distance (TTTG) of > 20 mm or < 8 mm, Caton-Deschamps Index of > 1.2 or < 0.8, lateral patellar tilt of > 5°, tibiofemoral malalignment defined as a mechanical valgus or varus of > 5°, femoral anteversion > 30° or tibial torsion of > 40° [12]. High-grade trochlear dysplasia was not a contraindication for PFIA.

### Characteristics of the prosthesis

The used prothesis is a cobalt chrome trochlear component connected to a titanium bone anchoring fixation stud via a taper interlock and an all-polyethylene patellar component. The implant is implanted flush with the surrounding cartilage in order to avoid the risk of patellofemoral overstuffing. The advantage of this prosthesis over earlier systems is a large lateralized offset to address cartilage defects in the lateral and superior parts of the trochlea. The craniolateral shape and deep plain bearing supply additional patella guiding between 0° and 30° knee flexion. This characteristic may be advantageous, especially in patients with femoral trochlear dysplasia or patellofemoral instability. Therefore, patellar maltracking with shear forces on the medial patellofemoral soft tissue and compressive loads on pathological native cartilage between the lateral trochlea and the lateral patella facet is thought to be minimized.

### Surgical technique

The detailed surgical technique has been described previously [5]. In short, the patient was positioned prone, and a tourniquet was attached. A lateral minimally invasive surgical approach without eversion of the patella was used to protect the medial soft tissue structures. With the knee in full extension, an offset drill guide was used to establish a working axis normal to the central trochlear articular surface and to confirm trochlear defect coverage. Once the superior and inferior drill guide feet were aligned with the trochlear orientation, a guide pin was advanced into the bone. The superior/inferior and the medial/lateral offsets were measured using specific instrumentation to determine the proper implant geometry. The implant bed was reamed three-dimensionally with the aid of a guide block. A special reamer was used to prepare the implant bed for the anterior preparation. The screw fixation stud was then advanced into the bone, and the trochlear component was aligned with the appropriate offsets on the implant holder and placed into the taper of the fixation stud. The trochlear component was then seated using an impactor. According to previous recommendations, the patella was routinely resurfaced in all patients with the inlay technique [14]. A drill guide was inserted with the aid of an alignment guide. The medial/lateral and superior/inferior offsets were measured, and an implant bed was reamed. The patellar component was then aligned on the implant holder and cemented into the bone bed.

### Postoperative rehabilitation

Postoperative rehabilitation started on the first postoperative day, focusing on range of motion and pain management. Full range of motion was allowed immediately. Partial weight bearing with 20 kg was recommended for two weeks, followed by a stepwise increase until full weight bearing was achieved. Moreover, rehabilitation included lymphatic drainage and continuous passive motion for the first two weeks. Patients were discharged from the hospital when they could bend the knee to 90 degrees and climb stairs on crutches.

### Outcome measures

All patients were evaluated preoperatively and at a minimum of 24 months postoperatively by an independent and trained examiner who was not involved in the patients’ treatment. Patient-reported outcomes (PROs) included the transformed Western Ontario and McMaster Universities Osteoarthritis Index (WOMAC score) [3] and the Visual Analogue Scale for Pain (VAS) [11]. For the WOMAC, standardized answer options were given as 5 Likert boxes, and each question scored 0–4. A normalized percentage score (100 indicating no problems and 0 indicating extreme problems) was calculated for each subscale of the WOMAC score (pain, stiffness, function).

To better quantify the level of improvement for the WOMAC total score it was evaluated how many of the patients reached the thresholds of the minimal clinically important difference (MCID) for overall PFIA [20]. For this aim the raw data (threshold − 8.5) [20] were transformed to the threshold of 8.9 according to the standardized algorithm out of the literature [3].

The Tegner Activity Scale [23] and a self-designed questionnaire, which assessed the number of pre- and postoperative sporting disciplines as well as sports frequency (defined as sessions per week) and sports duration (defined as hours per week), were used for sport-related outcomes [12].

Patient satisfaction with the procedure was assessed at 24 months follow-up. Postoperative complications and reoperations were recorded during the whole study period.

Radiographs included weight-bearing antero-posterior, lateral, and 30° patellar axial views. The Kellgren-Lawrence grading [15] and the Caton-Deschamps Index [4] were used to assess the progression of tibiofemoral OA and the differences in patellar height, respectively. Implant-related radiographic results compared the first to last follow-up radiographs assessing periprosthetic radiolucency, implant subsidence, cyst formation, and implant disassembly. Radiographic evaluation was performed using the Picture Archiving and Communication System (PACS, Philips Medical Systems, Sectra Imtec AB, Sweden).

### Statistical analysis

Data were analyzed using SPSS (v28.0, IBM-SPSS, New York, USA). Normally distributed data are reported as mean ± standard deviation, whereas non-normally distributed data are reported as median and range. For normally distributed data pre- and postoperative outcomes were compared using the parametric t-test. Statistical analysis was performed two-sided. The level of significance was set at *p* <.05.

A significant improvement on the transformed WOMAC total score of 24 points was determined in a previous study for patients following isolated PFIA [12]. According to these data, an a priori power analysis was calculated (two-tailed t-test) with a mean difference to detect 24 points and a standard deviation of 13.6 points in the WOMAC total score [12]. It established a sample size of a minimum of 5 patients with α = 0.05 for a power of 0.8 (G*Power 3.1.9.6, Düsseldorf, Germany) [8].

## Results

**Fig. 1:**
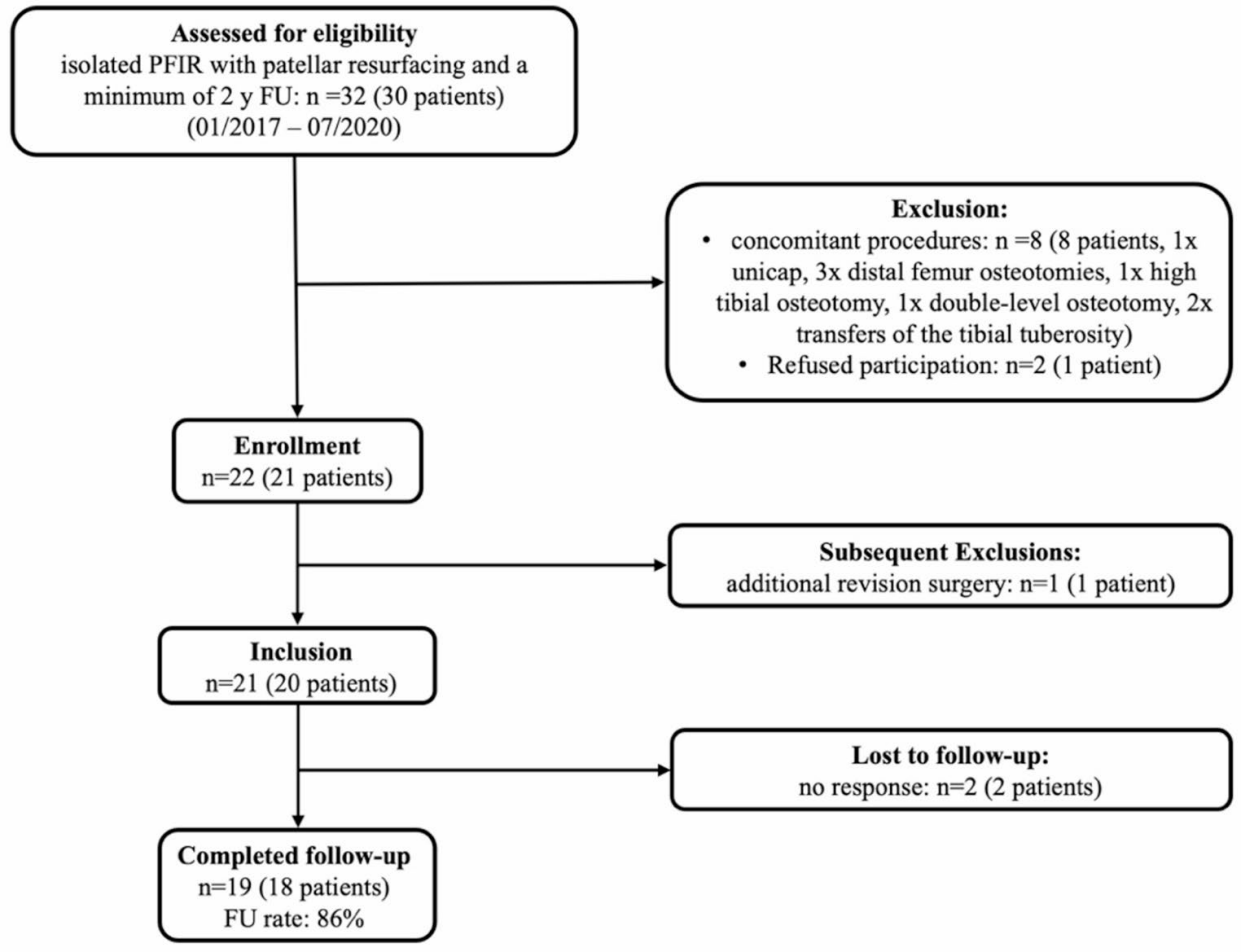
Flow chart for patient inclusion; n, number of knees; y, year; FU, Follow-up; Follow-Up rate is displayed as percentage of the total number of the enrolled knees

A total of 21 patients (22 knees) were eligible for inclusion, of which 18 patients (19 knees, 86%) were available for follow-up on average 28.2 ± 9.1 (range 24.0–55.0) months postoperatively (Fig. 1). Detailed patient characteristics and surgical history are shown in Table 1.

**Table 1:** Pre- and intraoperative characteristics of 18 patients

Number of knees, patients	19 (100%), 18
Gender distribution	
male	6 (31.6%), 6
female	13 (68.4%),12
Age (years)	50.9 ± 10.7
Body mass index (kg/m^2^)	26.0 ± 4.6
Surgical history of the patellofemoral joint (n)	7 (36.8%), 7
Debridement	1 (5.3%), 1
Microfracture	1 (5.3%),1
MACT	1 (5.3%),1
AOT	1 (5.3%),1
Trochleoplasty	2 (10.5),1
Transfer of tibial tuberosity	1 (5.3%),1
Trochlear dysplasia (Dejour classification)	
None	7 (36.8%), 7
Type A	4 (21.1%), 4
Type B	5 (26.3%), 5
Type C	2 (10.5%), 2
Type D	1 (5.3%),1

The first value is given as number of knees (percentage of the total number of knees), the second value is given as number of patients; mean ± standard deviation is given for Age and Body mass index of the patients at surgery; kg/m^2^, kilograms per square meter; AOT, autologous osteochondral transfer; MACT, Matrix-associated chondrocyte transplantation

### Patient-reported outcomes

Compared to preoperative levels, significant improvements were observed in VAS for pain, WOMAC total score, and all WOMAC sub scores, except for WOMAC stiffness, at final follow-up (Table 2). For the WOMAC total score 74% (*n* = 14) of the patients achieved the MCID threshold indicating meaningful improvement.

**Table 2:** Pre- and postoperative patient reported outcome measures

	Preoperative	2 Years	Delta	*p*
WOMAC total	55.8 ± 16.0 (48.7–63.0)	77.2 ± 17.0 (69.7–84.6)	21.3 ± 22.7 (11.1–31.5)	*p* <.001
WOMAC pain	56.1 ± 20.2 (47.1–65.0)	81.8 ± 17.0 (73.9–88.9)	25.8 ± 23.0 (15.5–36.1)	*p* <.001
WOMAC stiffness	57.2 ± 22.2 (47.4–67.1)	66.4 ± 28.9 (52.6–78.9)	9.2 ± 34.6 (-6.3–24.8)	*p* = .261
WOMAC function	55.6 ± 16.2 (48.5–62.9)	77.1 ± 16.9 (69.5–84.6)	21.4 ± 23.3 (11.0–31.9)	*p* <.001
VAS for pain^$^	7 (1–9)	3 (0–7)	3 (-1–8)	*p* <.001

Values are given as mean ± standard deviation and the 95% Confidence Interval; VAS, Visual Analogue Scale; $, displayed as median (range); p, significance level set to 0.05; Delta, describes the difference between preoperative und 2 Years values

Regarding overall postoperative satisfaction, 68% (*n* = 13) of patients reported being either very satisfied (*n* = 8, 42%) or satisfied (*n* = 5, 26%). Four patients (21%) were partially satisfied, while two patients (11%) expressed dissatisfaction due to persistent anterior knee pain during physical activities.

### Sports-related outcomes

**Fig. 2:**
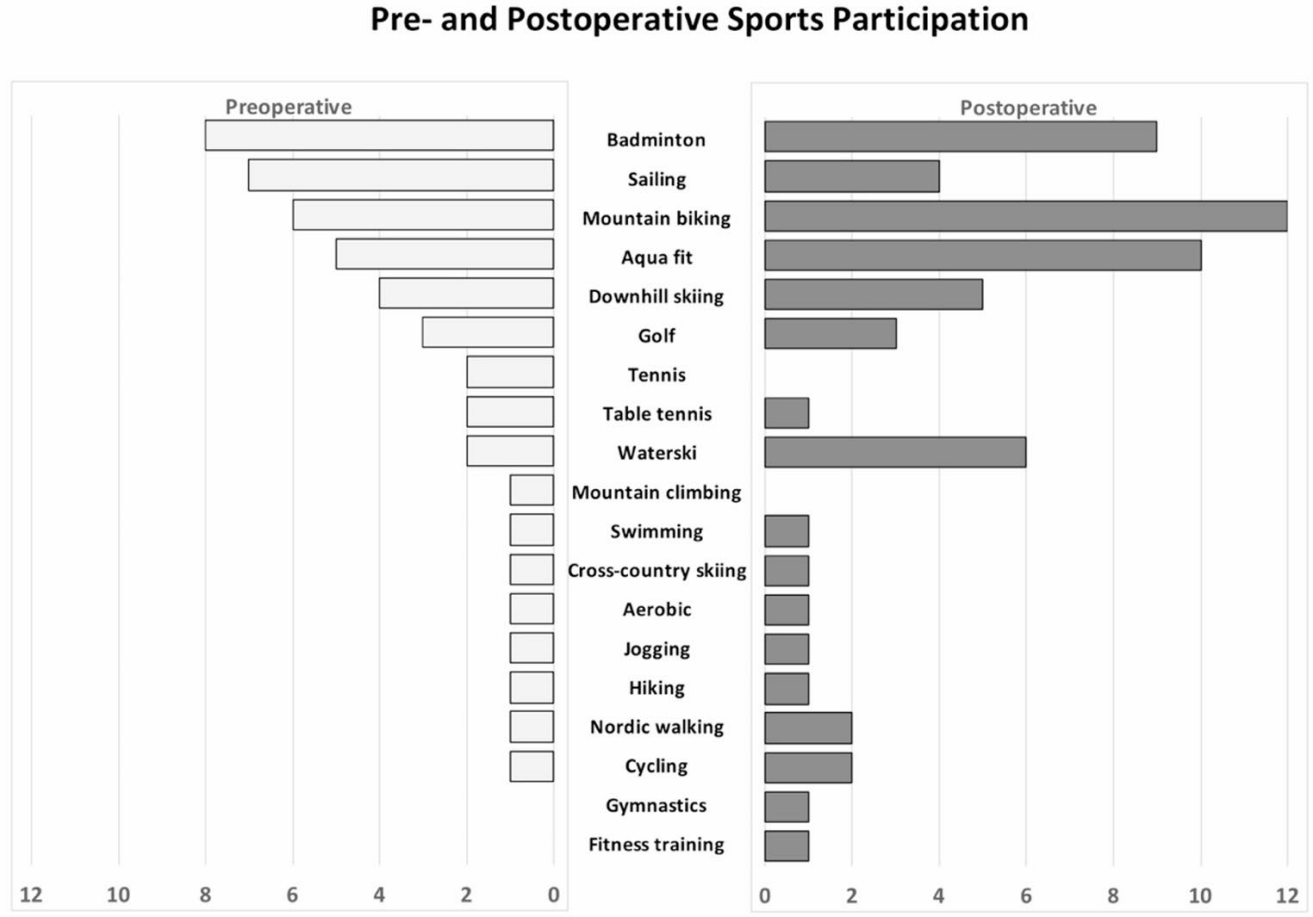
The number of sports disciplines in which patients participated one year before and 24 months after surgery. In some patients, more than one discipline was performed

Fourteen patients (78%) participated in sporting activities preoperatively, which increased to 17 patients (94.4%) postoperatively (*p* = .14). There were no significant changes in the frequency or duration of activity (Table 3). Figure 2 shows all sports disciplines in which patients participated one year before and 24 months after surgery.

**Table 3:** Sports-related results

	Preoperative	2 Years	*p*
Number of Sports disciplines	1.7 ± 2.2 (0–8)	1.9 ± 3.2 (0–12)	
Sports frequency (sessions per week)	2.4 ± 2.3 (0–7)	2.4 ± 1.3 (0–5)	*p* = .979
Sports duration (hours per week)	3.7 ± 4.0 (0–15)	3.2 ± 2.7 (0–10)	*p* = .422
Tegner Activity Scale^$^	2 (1–5)	3 (1–6)	*p* = .110

Values are given as mean ± standard deviation and range; $, displayed as median (range); p, significance level set to 0.05

A slight but statistically insignificant improvement was observed in the Tegner Activity Scale. In detail, nine patients (47%) experienced an increase in activity level, six patients (32%) returned to their preoperative activity level and four patients (21%) did not regain their preoperative activity level (Table 3).

### Radiographic outcomes

There was no significant progression of tibiofemoral OA among the patient cohort (Table 4). Furthermore, no significant changes in patellar height or any evidence of periprosthetic loosening, cyst formation, or implant subsidence were observed.

**Table 4:** Radiographic results

	Preoperative	2 Years	*p*
tibiofemoral KL -grade^§^	0: 5 (26.3) 1: 6 (31.6) 2: 7 (36.8) 3: 1 (5.3)	0: 4 (21.1) 1: 7 (36.8 2: 7 (36.8) 3: 1 (5.3)	*p* = .317
Caton-Deschamps Index	0.9 ± 0.1	1.2 ± 0.9	*p* = .647

KL, Kellgren-Lawrence grading; §, Values displayed as number (%); Caton-Deschamps Index displayed as mean ± standard deviation, p, significance level set *p* = .05

### Complications

No intraoperative complications occurred. One patient (5.3%) required revision surgery due to aseptic loosening, probably caused by the initial choice of a too-small implant. Revision surgery with a larger trochlear component size was performed 13 months after the index surgery.

## Discussion

The primary finding of the present study was that isolated PFIA using an inlay trochlear component featuring an enlarged lateral offset provided favorable postoperative outcomes with significant improvements in pain and knee function at short-term follow-up. Failure- and complication rates were low, including those for implant-related maltracking (catching, snapping, clunking) or progression of tibiofemoral OA.

In the case of symptomatic end-stage patellofemoral OA, PFIA is a valuable treatment option for unicompartmental joint replacement [12, 14]. However, PFIA is often associated with complications such as patellofemoral maltracking and instability, which can lead to secondary OA. Recent studies have suggested that inlay implants should be considered contraindicated in patients with patella alta or with a craniolateral type of arthritis with additional lateralization, as commonly seen in a dysplastic trochlea [1, 14]. The primary limitation of current PFIA models lies in their inability to address these specific anatomical challenges. Existing designs do not cover the proximal and lateral areas of the trochlea, instead focusing on restoring normal anatomy in the central region flush with surrounding cartilage. Furthermore, they do not account for sagittal rotational correction or coronal alignment. As a result, in cases of dysplastic trochlea with even slight rotational errors of the femur, the implant is often positioned in an internally rotated orientation. This leaves the proximal dysplastic anatomy uncorrected, leading to patellar maltracking during early flexion. As a result, a supraphysiological peak pressures on the native pathological cartilage between the lateral trochlea and the lateral patella facet developed. Over time, this resulted in cartilage abrasion with pain and clunking. To date, onlay implants have been superior in addressing these problems [2, 17]. However, onlay prostheses, compared to inlay implants, may overstuff the patellofemoral joint and have been associated with higher rates of progression in tibiofemoral osteoarthritis [9, 21].

To mitigate these limitations, the design of the modified prosthesis aimed to combine the advantages of an inlay prosthesis (no overstuffing) with those of an onlay implant (static patella guiding in early flexion) by incorporating an enlarged lateral offset. In the current study, no failures related to pain or implant-induced patellar maltracking (such as catching, snapping, or clunking) were observed, suggesting that this new design successfully guides the patella in terminal extension while avoiding excessive loads on the native cartilage between the lateral trochlea and patellar facet.

The implantation of this trochlea component featuring an enlarged lateral offset might therefore offer a favorable solution for patients with patella alta or a dysplastic trochlea.

Regarding the indication for concomitant procedures alongside PFIA, established treatment guidelines exist for patients with concomitant trochlea dysplasia, patellofemoral instability, malalignment, or patella alta [10, 12]. However, previous studies have shown that concomitant procedures addressing patellofemoral instability or malalignment are significantly correlated with early postoperative failures [12, 14]. These failures may be attributed to the limited possibility of traditional PFIA implants to correct patellofemoral maltracking making even additional procedures insufficient to restore the physiological patellofemoral tracking necessary for optimal biomechanics. In the present study, no cases of patellar instability associated with medial soft-tissue injury or clinical failure – defined as a WOMAC total score below 43, were observed at final follow up [25]. It seems, that the enlarged trochlea design protects the medial soft tissues by preventing lateralized patella maltracking.

This study provides the first outcome data for a newly designed inlay prosthesis. Previously published PROs following isolated implantation of latest generation inlay and onlay systems at a two-year follow-up (WOMAC total score of 78) [9] are comparable to those reported in the present study. These findings suggest that the newly designed prosthesis is a viable option for isolated PFIA.

Patients with patellofemoral OA are typically relatively young and have therefore high expectations for functional outcomes, including the ability to return to sports [16, 22]. Compared to other commonly used PFIA systems, the sports participation rates, Tegner Activity Scale, and the mean number of sports sessions per week reported at the two-year follow-up are comparable to those reported in the present study [12]. This suggests that the new arthroplasty design allows patients to return to the same level of sports activity as conventional inlay arthroplasty.

Although only sports activity with a moderate risk profile are recommended after patellofemoral arthroplasty [7], a few of the patients in this study participated in higher-risk pivoting sports like badminton (Fig. 2). In these cases, the new design with its enlarged lateral expression may offer advantages by preventing patella subluxations. This is achieved through efficient static patella guidance during unanticipated change in direction and lateral shear forces acting on the knee. Whether high-risk sports participation affects long-term survival of PFIA is currently unknown and requires further investigation.

Recent studies have reported a higher rate of OA progression with onlay designs compared to inlay designs at short and medium follow-ups, mainly attributed to patellar catching, anterior notching, or patellofemoral overstuffing [2, 9, 21]. In contrast, Familiari et al. reported a higher rate of revision to TKA 9 years after PFIA compared to patellofemoral onlay arthroplasty after 4 years due to instability, pain, malposition, stiffness and wear of the patellar component [6]. In the present study, none of these failures nor any significant radiographic OA progression was observed at the 2-year follow-up. Whether the inlay trochlear component with an enlarged lateral offset prevents tibiofemoral OA over a longer follow-up period remains unknown. To investigate this question, our database will continue to monitor these patients for an extended follow-up to build on these preliminary results.

### Limitations

This study is not without limitations. First, the patient number was relatively small, however, it is comparable to other reports on this topic [1, 9, 12, 26]. Second, the study’s non-comparative nature at a single institution needs to be highlighted. Further studies with a longer follow-up will be necessary to confirm the present results, to determine potential risk factors for failure and to assess the progression of OA. Despite these limitations, the results of this study suggest that the novel prosthesis can be considered a viable alternative to currently available inlay and onlay designs. By combining the potential advantages of both designs, this prosthesis appears particularly suitable in patients with patella alta or with a proximal and lateral type of arthritis, as is often observed in cases of trochlear dysplasia.

## Conclusion

Isolated PFIA utilizing an implant with an enlarged lateral offset has been shown to be an effective and safe intervention for patients with symptomatic patellofemoral OA. The procedure significantly improved knee function and pain relief, with low failure rates observed at short-term follow-up.
