# Correction: Improved clinical outcomes and a low rate of failure following implantation of a patellofemoral inlay arthroplasty model featuring an enlarged lateral offset– a prospective clinical and radiographic evaluation at short term follow-up

**DOI:** 10.1007/s00402-025-05931-8

**Published:** 2025-06-07

**Authors:** Matthias Cotic, Tiago Martinho, Svenja Höger, Marco-Christopher Rupp, Maximilian Hinz, Sebastian Siebenlist, Andreas B. Imhoff, Armin Runer

**Affiliations:** 1 Department of Sports Orthopaedics, Technical University of Munich, Ismaninger Str. 22, 81675 Munich, Germany; 2 Faculty of Medicine, University of Geneva, Geneva, Switzerland

**Archives of Orthopaedic and Trauma Surgery (2025) 145:208**

10.1007/s00402-025-05832-w

In the original version of this article, Author contributions section, Funding section, Acknowledgments section, Conflict of interest, Ethical approval and Informed consent were missing and should have been.

**Author contributions** MC and AR designed the study. MC collected data. MC provided ethical approval and wrote the manuscript. AR performed the statistical analysis. MC, TM, SH, MH, MR, and SS provided figures and made edits to the manuscript. ABI and AR conceived of the study helped with data interpretation, and critically reviewed the manuscript. All authors read and approved the final manuscript.

**Funding** The study was not sponsored.

**Acknowledgments** The authors thank Eva Bartsch, BSc for her valuable work in data collection.

**Conflict of interest** Andreas B. Imhoff is a consultant for Arthrosurface, Franklin, MA, USA. However, the company had no impact on the study design, the results, or the writing of the manuscript. Sebastian Siebenlist has received consultant fee payments from Arthrex GmbH, KLS Martin Group and medi GmbH & Co. KG unrelated to this study.

**Ethical approval** The study was approved by the institutional review board of the Technical University of Munich (reference: 2022-200-S), and the study was performed in accordance with the Declaration of Helsinki.

**Informed consent** Written informed consent was obtained from all patients.

